# Impact of MCA stenosis on the early outcome in acute ischemic stroke patients

**DOI:** 10.1371/journal.pone.0175434

**Published:** 2017-04-07

**Authors:** Jiann-Shing Jeng, Fang-I Hsieh, Hsu-Ling Yeh, Wei-Hung Chen, Hou-Chang Chiu, Sung-Chun Tang, Chung-Hsiang Liu, Huey-Juan Lin, Shih-Pin Hsu, Yuk-Keung Lo, Lung Chan, Chih-Hung Chen, Ruey-Tay Lin, Yu-Wei Chen, Jiunn-Tay Lee, Chung-Hsin Yeh, Ming-Hui Sun, Ta-Chang Lai, Yu Sun, Mu-Chien Sun, Po-Lin Chen, Tsuey-Ru Chiang, Shinn-Kuang Lin, Bak-Sau Yip, Chin-I Chen, Chi-Huey Bai, Sien-Tsong Chen, Hung-Yi Chiou, Li-Ming Lien, Chung Y. Hsu, on behalf of Taiwan Stroke Registry Investigators

**Affiliations:** 1 Department of Neurology, National Taiwan University Hospital, Taipei, Taiwan; 2 School of Public Health, College of Public Health, Taipei Medical University, Taipei, Taiwan; 3 Department of Neurology, Shin Kong Wu-Ho-Su Memorial Hospital, Taipei, Taiwan; 4 Department of Neurology, China Medical University Hospital, Taichung, Taiwan; 5 Department of Neurology, Chi Mei Medical Center, Tainan, Taiwan; 6 Department of Neurology, E Da Hospital, Kaohsiung, Taiwan; 7 Section of Neurology, Kaohsiung Veterans General Hospital, Kaohsiung, Taiwan; 8 Department of Neurology, Taipei Medical University-Shuang Ho Hospital, New Taipei City, Taiwan; 9 Department of Neurology, National Cheng Kung University Hospital, Tainan, Taiwan; 10 Department of Neurology, Kaohsiung Medical University Hospital, Kaohsiung, Taiwan; 11 Department of Neurology, Landseed Hospital, Taoyuan, Taiwan; 12 Department of Neurology, Tri-Service General Hospital, Taipei, Taiwan; 13 Department of Neurology, Yuan Rung Hospital, Yuanlin Township, Changhua, Taiwan; 14 Department of Neurology, Kuang Tien General Hospital, Taichung, Taiwan; 15 Department of Neurology, Cheng-Hsin General Hospital, Taipei, Taiwan; 16 Department of Neurology, En Chu Kong Hospital, New Taipei City, Taiwan; 17 Department of Neurology, Changhua Christian Hospital, Changhua, Taiwan; 18 Department of Neurology, Taichung Veterans General Hospital, Taichung, Taiwan; 19 Department of Neurology, Cathay General Hospital, Taipei, Taiwan; 20 Department of Neurology, Buddhist Tzu Chi General Hospital Taipei Branch, New Taipei City, Taiwan; 21 Department of Neurology, National Taiwan University Hospital, Hsinchu Branch, Hsinchu, Taiwan; 22 Department of Neurology, Taipei Medical University-Wan Fang Hospital, Taipei, Taiwan; 23 Department of Public Health, College of Medicine, Taipei Medical University, Taipei, Taiwan; 24 Department of Neurology, Chang Gung Memorial Hospital, Linkou, Taiwan; 25 Department of Neurology, College of Medicine, Taipei Medical University, Taipei, Taiwan; 26 Graduate Institute of Clinical Medical Science, China Medical University and Hospital, Taichung, Taiwan; "INSERM", FRANCE

## Abstract

**Background:**

Asians have higher frequency of intracranial arterial stenosis. The present study aimed to compare the clinical features and outcomes of ischemic stroke patients with and without middle cerebral artery (MCA) stenosis, assessed by transcranial sonography (TCS), based on the Taiwan Stroke Registry (TSR).

**Methods:**

Patients with acute ischemic stroke or transient ischemic attack registered in the TSR, and received both carotid duplex and TCS assessment were categorized into those with stenosis (≥50%) and without (<50%) in the extracranial internal carotid artery (ICA) and MCA, respectively. Logistic regression analysis, Kaplan-Meier method and Cox proportional hazard model were applied to assess relevant variables between groups.

**Results:**

Of 6003 patients, 23.3% had MCA stenosis, 10.1% ICA stenosis, and 3.9% both MCA and ICA stenosis. Patients with MCA stenosis had greater initial NIHSS, higher likelihood of stroke-in-evolution, and more severe disability than those without (all *p*<0.001). Patients with MCA stenosis had higher prevalence of hypertension, diabetes and hypercholesterolemia. Patients with combined MCA and extracranial ICA stenosis had even higher NIHSS, worse functional outcome, higher risk of stroke recurrence or death (hazard ratio, 2.204; 95% confidence intervals, 1.440–3.374; *p*<0.001) at 3 months after stroke than those without MCA stenosis.

**Conclusions:**

In conclusion, MCA stenosis was more prevalent than extracranial ICA stenosis in ischemic stroke patients in Taiwan. Patients with MCA stenosis, especially combined extracranial ICA stenosis, had more severe neurological deficit and worse outcome.

## Introduction

Intracranial arterial stenosis (ICAS) is increasingly recognized as an important factor in defining stroke subtypes and in selecting preventive or therapeutic measures [1–6]. Patients with symptomatic ICAS may have a higher risk of recurrent stroke [1–7]. The prevalence of ICAS among stroke patients varies across ethnic groups [3,4,8–15]. Asians, Africans, and Hispanics have a greater preponderance for ICAS than Caucasians [11–17]. ICAS is noted in 5% to 10% of Caucasians patients with ischemic strokes [10,15–19], but in 28% to 54% in Asian counterparts [11–14]. In Chinese patients with ischemic stroke, the middle cerebral artery (MCA) was the most commonly identified location of ICAS [19].

The Taiwan Stroke Registry (TSR) is a prospective, multicenter registry of patients with stroke or transient ischemic attack (TIA) [20]. The aims of the TSR are to investigate the risk factors, stroke types, and outcome in a nation-wide stroke registry, and to assess the quality of stroke care. The present study aimed to compare the risk factors, clinical features and outcome between ischemic stroke patients with and without MCA stenosis, assessed by transcranial sonography (TCS), based on the Taiwan Stroke Registry (TSR) database. The impact of superimposed extracranial internal carotid artery (ICA) stenosis was also assessed.

## Materials and methods

### Patients

Patients with acute ischemic stroke or TIA who were admitted to 39 TSR hospitals constitute the TSR cohort [20]. The diagnosis of ischemic stroke and TIA was acute neurologic dysfunction of vascular origin lasting for more and less than 24 hours, respectively. All patients received examination including computed tomography (CT) or/and magnetic resonance imaging (MRI) for the index event. They were included in the present study if they met the following criteria: (1) acute ischemic stroke or TIA; (2) receiving both duplex and TCS assessment; and (3) having been followed for at least 3 months. This study was approved by the Institutional Review Board of Taipei Medical University. The TSR protocols and the consent procedure were approved by the Institutional Review Board of each participating hospital, and the registered stroke patients gave their written informed consent for follow-up. All clinical investigation conducted according to the principles expressed in the Declaration of Helsinki.

TSR provides a structured record of demographics, including risk factors, stroke types, stroke subtypes (TOAST classification), National Institute of Health Stroke Scale (NIHSS) on admission and discharge, neuroimaging and ultrasonographic examination of relevant arteries, in-hospital management and complications, and functional outcomes in follow-up [20].

### Ultrasonographic study

Extracranial ICA and MCA stenosis were diagnosed by carotid duplex and TCS, respectively, and were categorized into <50% and ≥50% stenosis according to the following criteria. Extracranial ICA stenosis ≥50% was recorded if the peak systolic velocity of the ICA ≥125 cm/s, or the peak systolic velocity ratio of the ICA to the ipsilateral common carotid artery ≥2, or no Doppler flow signal detected in the ICA indicating total occlusion [21,22]. MCA stenosis ≥50% was recorded if the peak systolic velocity (PSV) ≥140 cm/s, mean flow velocity (MFV) ≥100 cm/s, only trickle flow signals, or no detectable Doppler flow signal in the MCA in patients with a good temporal window [19,23–27]. Patients with poor temporal bone windows were excluded. These criteria were determined by a panel of neurosonographic experts based on published criteria [23,24]. We have validated the PSV criteria in diagnosis of MCA stenosis ≥50% [25,26]. The accuracy of MFV criteria in diagnosis of MCA stenosis was reported [27], and further validated in recent studies in Asians [28]. All sonographic studies were reimbursed by National Health Insurance (NHI), the universal payer in Taiwan, under strict guidelines. Ultrasonographic assessment was conducted by trained neurosonographers who have gone through strict certification processes to be qualified for sonographic claims for NHI reimbursement.

### Patient follow-up

All registered stroke patients were followed in clinics at 1 and 3 months poststroke. For those who could not attend the clinic, telephone interviews were made to assess functional status, and to check for stroke recurrence and survival status. In the present study, functional outcome 3 months after stroke was assessed using modified Rankin Scale (mRS) and Barthel Index. A mRS of 2 or greater at 3 months was considered an unfavorable outcome.

### Statistical analysis

Categorical variables are presented as percentage and continuous or discrete variables mean ± standard deviation or median (25th-75th percentile). Student’s *t*-test, χ^2^ test, ANOVA and Kruskal-Wallis test were used for univariate analysis between groups with relevant variables as indicated. A logistic regression analysis was adapted to evaluate the odds ratio and 95% confidence intervals (CI) of predictors of MCA stenosis. The Kaplan-Meier method was used to estimate the probability of early stroke recurrence and mortality 3 months after stroke. The Cox proportional hazards model was used for the hazard ratio (HR) and 95% CI of predictors of early stroke recurrence and mortality. Statistical analysis was performed at an α level of 0.05 (two-tailed). SAS (SAS Institute, Cary, NC, release 9.1) was used for statistical analyses.

## Results

Of 29,195 TSR stroke patients [20], 6,757 received both duplex and TCS assessment, with 754 (11.2%) patients who had poor temporal bone window excluded from this study. Thus, 6,003 patients (male, 75.6%; average age, 65.3±12.9 years) fulfilled the selection criteria for the present study. Of the 6,003 patients, 5,430 (91.5%) were ischemic stroke and 573 (8.5%) were TIA. Of these, 1,397 (23.3%) had MCA stenosis ≥50%, 608 (10.1%) ICA stenosis ≥50%, and 236 (3.9%) both MCA and ICA stenosis ≥50%. The frequency of MCA stenosis was 2.3-fold higher than that of ICA stenosis. Of 608 patients with ICA stenosis, 183 (30.1%) had ipsilateral MCA stenosis, and 53 (8.7%) had MCA stenosis on the contralateral side.

Patients with MCA stenosis had significantly higher frequencies of hypertension (81.2% vs 76.6%), diabetes mellitus (DM) (46.1% vs. 35.6%), ischemic heart disease (13.7% vs 11.7%), hypercholesterolemia (44.6% vs. 38.4%) and hypertriglyceridemia (33.1% vs 26/7%) than those without MCA stenosis. Large-artery atherosclerosis (55.9%) was the most common stroke subtype and small-vessel occlusion (22.7%) was the second in those with MCA stenosis. However, among those without MCA stenosis, the most common stroke subtype was small-vessel occlusion (50.1%) and the second was large-artery atherosclerosis (23.2%). The distribution of cardioembolism, specific etiology, and undetermined etiology were similar between patients with and without MCA stenosis. The treatment of antiplatelets and carotid stenting were almost the same among patents with and without MCA stenosis. There was around 80% of patients with antiplatelets and only 0.2% with carotid stenting (Table 1). The multivariate analysis showed only DM (OR, 1.55; 95% CI, 1.33–1.81), hypercholesterolemia (OR, 1.22; 95% CI, 1.05–1.43) and family history of stroke (OR, 1.22; 95% CI, 1.04–1.44) were significantly related to MCA stenosis (Table 2).

**Table 1:** Comparison between patients with or without MCA stenosis.

	All	MCA <50%	MCA ≥50%	*P* value
	(n = 6003)	(n = 4606)	(n = 1397)	
Male sex	4537 (75.6%)	3470 (75.3%)	1067 (76.4%)	0.427
Mean age, y	65.3±12.9	65.2±12.9	65.7±12.7	0.255
Body mass index, kg/m^2^	24.6±3.7	24.6±3.7	24.6±3.7	0.755
***Risk factors***				
Hypertension	4663 (77.7%)	3529 (76.6%)	1134 (81.2%)	<0.0001
Diabetes mellitus	2286 (38.1%)	1642 (35.6%)	644 (46.1%)	<0.0001
Ischemic heart disease	732 (12.2%)	540 (11.7%)	192 (13.7%)	0.043
Atrial fibrillation	656 (10.9%)	510 (11.1%)	146 (10.5%)	0.514
Hypercholesterolemia	2394 (39.9%)	1771 (38.4%)	623 (44.6%)	<0.0001
Hypertriglyceridemia	1693 (28.2%)	1230 (26.7%)	463 (33.1%)	<0.0001
Cigarette smoking	3070 (51.1%)	2367 (51.4%)	703 (50.3%)	0.484
Alcohol drinking	1098 (18.3%)	852 (18.5%)	246 (17.6%)	0.452
Family history of stroke	1444 (24.1%)	1082 (23.5%)	362 (25.9%)	0.064
***Treatment***				
Antiplatelets	4827 (80.4%)	3711 (80.6%)	1116 (79.9%)	0.573
Carotid stenting	10 (0.2%)	7 (0.2%)	3 (0.2%)	0.614
***Stroke types***				0.002
Transient ischemic attack	573 (8.5%)	469 (10.2%)	104 (7.4%)	
Ischemic stroke	5430 (91.5%)	4137 (89.8%)	1293 (92.6%)	
Stroke subtype (TOAST)				< .0001
LAA	1681 (31.0%)	958 (23.2%)	723 (55.9%)	
SVO	2366 (43.6%)	2072 (50.1%)	294 (22.7%)	
CE	485 (8.9%)	393 (9.5%)	92 (7.1%)	
SE	80 (1.5%)	61 (1.5%)	19 (1.5%)	
UE	818 (15.1%)	653 (15.8%)	165 (12.8%)	
***Clinical course***				
Initial NIHSS	4 (2–7)	4 (2–7)	5 (3–9)	<0.001
Stroke in evolution	413 (6.9%)	289 (6.3%)	124 (8.9%)	0.001
mRS at 3 m	2 (1–3)	1 (1–3)	2 (1–4)	<0.001
mRS ≥2 at 3 m	3096 (51.6%)	2247 (48.8%)	849 (60.8%)	<0.001

Values are numbers with percentage in parenthesis for each category except for age and body mass index that are presented as mean ± standard deviation. Stroke subtypes were based on TOAST (Trial of Org 10172 in Acute Stroke Treatment) classification; LAA, large-artery atherosclerosis; SVO, small-vessel occlusion; CE, Cardioembolism; SE, specific etiology; UE, undetermined etiology.

**Table 2:** Multivariate analysis of factors related to MCA stenosis.

	Odds ratio	95% Confidence interval	p value
Male sex	1.08	0.88–1.32	0.459
Mean age, y	1.00	0.99–1.01	0.851
Body mass index, kg/m^2^	1.00	0.98–1.02	0.815
Hypertension	1.10	0.91–1.34	0.318
Diabetes mellitus	1.55	1.33–1.81	0.001
Ischemic heart disease	1.16	0.94–1.44	0.174
Atrial fibrillation	1.04	0.81–1.32	0.772
Hypercholesterolemia	1.22	1.05–1.43	0.011
Hypertriglyceridemia	0.93	0.78–1.11	0.434
Cigarette smoking	0.97	0.82–1.16	0.758
Alcohol drinking	0.93	0.76–1.14	0.965
Family history of stroke	1.22	1.04–1.44	0.013

Patients with MCA stenosis had higher NIHSS on presentation, more likely to have stroke in evolution, worse functional status (Barthel Index and mRS) and higher mortality or stroke recurrence at 3 months after stroke than those without MCA stenosis. Unfavorable outcome with a mRS ≥2 at 3 months was higher in patients with MCA stenosis than those without (60.8% vs. 48.8%, p<0.0001) (Table 1). Patients with combined MCA and ICA stenosis had even higher NIHSS on presentation, worse functional outcome, and higher mortality or stroke recurrence at 3 months than those with MCA stenosis only (Table 3).

**Table 3:** Comparison among patients with or without stenosis of the MCA and ICA.

	MCA <50%		MCA ≥50%		*P* value
	ICA<50%	ICA≥50%	ICA<50%	ICA≥50%	
	(n = 4234)	(n = 372)	(n = 1161)	(n = 236)	
Male sex	3151 (74.4%)	319 (85.8%)	870 (74.9%)	197 (83.5%)	<0.001
Mean age, y	64.7±12.9	70.6±11.9	64.9±12.9	69.7±11.2	<0.001
Hypertension	3225 (76.2%)	304 (81.7%)	947 (81.6%)	187 (79.2%)	<0.001
Diabetes mellitus	1512 (35.7%)	130 (34.9%)	547 (47.1%)	97 (41.1%)	<0.001
Ischemic heart disease	465 (11.0%)	75 (20.2%)	138 (11.9%)	54 (22.9%)	<0.001
Atrial fibrillation	465 (11.5%)	45 (12.1%)	116 (10.0%)	30 (12.7%)	0.389
Hypercholesterolemia	1630 (38.5%)	141 (37.9%)	521 (44.9%)	102 (43.2%)	<0.001
Hypertriglyceridemia	1001 (23.6%)	68 (18.3%)	309 (26.6%)	48 (20.3%)	0.005
Cigarette smoking	2125 (50.2%)	242 (65.1%)	564 (48.6%)	139 (58.9%)	<0.001
Alcohol drinking	781 (18.4%)	71 (19.1%)	209 (18.0%)	37 (15.7%)	0.713
Antiplatelets	3419 (80.8%)	292 (78.5%)	930 (80.1%)	186 (78.8%)	0.653
Carotid steting	0 (0.0%)	7 (1.9%)	0 (0.0%)	3 (1.3%)	< .001
Initial NIHSS score	4 (2–7)	5 (3–10)	5 (2–9)	6 (3–12)	<0.001
Stroke in evolution	246 (5.8%)	43 (11.6%)	99 (8.5%)	25 (10.6%)	<0.001
mRS at 3 m	1 (0–3)	2 (1–4)	2 (1–3)	3 (1–4)	<0.001
Barthel index at 3 m	100 (80–100)	95 (55–100)	100 (70–100)	90 (30–100)	<0.001
Death or stroke recurrence at 3 m	173 (4.1%)	27 (7.3%)	68 (5.9%)	24 (10.2%)	<0.001

NIHSS, National Institute of Health Stroke Scale; mRS, modified Rankin Scale. Values are median (interquartile range) for NIHSS, Barthel index and mRS; and number (percentage) for other categories.

**Fig 1:**
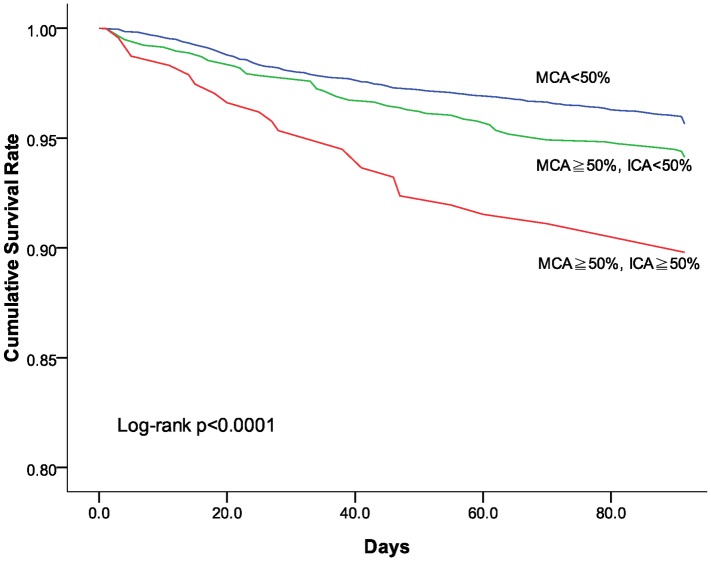
Cumulative survival (free of recurrent stroke or death) among patients with or without MCA stenosis.

The Kaplan-Meier analysis based on stroke recurrence or mortality at 3 months shows patients without MCA stenosis fared better with lower rate of stroke recurrence or death than those with MCA stenosis (log rank p<0.001). Patients with both MCA and ICA had even worse outcome than those with MCA stenosis based on stroke recurrence rate and mortality at 3 months poststroke (Fig 1). The Cox multivariate regression model also demonstrated that ischemic stroke patients with both MCA and ICA stenosis had higher stroke recurrence or death than those without MCA stenosis (HR, 2.204; 95% CI, 1.440–3.374; p<0.001) (Table 4).

**Table 4:** Cox-model for stroke recurrence or death at 3 months after stroke.

	All patients		Ischemic stroke patients	
	(n = 6003)		(n = 5430)	
	HR (95% CI)	p value	HR (95% CI)	p value
Male sex	1.316 (0.984–1.759)	0.064	1.380 (1.019–1.867)	0.037
Mean age, y	1.028 (1.018–1.038)	<0.001	1.029 (1.019–1.040)	<0.001
Diabetes mellitus	1.258 (1.004–1.577)	0.046	1.271 (1.008–1.602)	0.043
MCA <50%	1.000	—	1.000	—
MCA ≥50%				
ICA <50%	1.344 (1.019–1.771)	0.036	1.287 (0.966–1.713)	0.085
ICA ≥50%	2.204 (1.440–3.374)	<0.001	2.154 (1.406–3.298)	<0.001

HR indicates hazard ratio; CI, confidence interval.

## Discussion

This study was based on the TSR [20], a nation-wide, large-scale stroke registry with rigorous control of entry data to compare selective clinical features and outcomes between ischemic stroke patients with and without MCA stenosis. Results show that MCA stenosis was more frequently found than extracranial ICA stenosis in ischemic stroke patients. The MCA stenosis to ICA stenosis (23.3% vs. 10.1%) ratio of 2.3 is in agreement with studies in other Asian populations [12,13,15,19].

Coexisting ICA and MCA stenosis were found in 3.9% of patients in the present study. Among patients with extracramial ICA stenosis, 38.6% had MCA stenosis. In the North American Symptomatic Carotid Endarterectomy Trial, one-third of patients with symptomatic extracranial ICA stenosis also had ICAS [29]. In a Hong Kong study, 10% of patients with ischemic stroke had both intracranial and extracranial lesions [19]. ICAS was noted in 48% of patients with extracranial carotid stenosis in Korea [30]. The findings suggest that in patients with extracranial carotid stenosis, ICAS is a common comorbidity and should be searched for. This situation is especially important for Asian patients, who have higher prevalence rate of ICAS [11–16], and are considered for carotid endarterectomy or stenting. ICAS is known to unfavorably affect the outcome following surgical or stenting interventions on carotid artery stenosis [29,30].

The present study also shows that patients with MCA stenosis presented with higher NIHSS, which reflects more severe neurological deficit at stroke onset, were more likely to manifest stroke-in-evolution and to have unfavorable outcome and higher mortality at 3 months after stroke as compared to patients without MCA stenosis. Within the MCA stenosis group, those with superimposed ICA stenosis had even higher NIHSS on admission, worse outcome, and higher mortality than those without ICA stenosis. In a survival analysis, the endpoint of combined stroke recurrence or death was significantly increased in patients with both MCA and increased further among those with both MCA and ICA stenosis [31].

The Warfarin-Aspirin Symptomatic Intracranial Disease (WASID) study showed that the annual recurrence rates of stroke or TIA with symptomatic intracranial atherosclerosis were nearly 20%, and most subsequent strokes were in the same territory and nonlacunar [32]. Higher recurrence rates of ischemic events have also been reported in patients with symptomatic MCA disease compared to those who were asymptomatic [33]. In the subgroup of our patients with MCA≥50%/ICA<50%, symptomatic MCA stands for 45.9%. In the subgroup of our patients with MCA ≥50%/ICA ≥50%, symptomatic MCA stands for 58.7%. The percentage of symptomatic MCA in the index stroke was around 46%-59% in our study. Since symptomatic MCA stenosis predicts worse outcome, more rapid and rigorous preventive measures would seem warranted for this patient population. A recent study suggests that more rigorous interventions may be warranted in patients with symptomatic ICAS [5,6]. MCA stenosis may be asymptomatic to the index stroke. Asymptomatic ICAS is often relatively benign. Among the WASID patients, 27.3% had asymptomatic ICAS, and the 1-year risk of stroke in the asymptomatic ICAS territory was 3.5% [34].

Results from the present study show that there was a difference in risk factor profile between patients with and without MCA stenosis. Patients with MCA stenosis had significantly higher rates of DM and hypercholesterolemia. DM has a significant impact on the extent of ICAS [35,36]. In one study of asymptomatic high-risk patients in Hong Kong, DM was linked to increased risk of MCA stenosis [19]. The present study also demonstrates that hypercholesterolemia was more frequently noted in patients with MCA stenosis than those without. In the WASID study, hypercholesterolemia is a determinant of ICAS with an odds ratio of 1.62 [37].

The strengths of the present study include the prospective, multicenter stroke registry based on a well-designed registry protocol with rigorous quality control [20]. The large sample size provides useful profiles to broaden our view into subpopulations of patients with ischemic stroke. Results derived from the present study support the contention that MCA stenosis is a marker for more severe neurological deficit at stroke onset, worse functional outcome, higher stroke recurrence rate or mortality. Although TCS has been widely applied to stroke patients its benefit have yet to be fully established [38]. In patients with TIA, abnormal TCS findings were noted to be useful in predicting subsequent cardiovascular events [17,39]. The multispecialty panel of experts convened by the Clinical Practice Committee of the American Society of Neuroimaging has defined TCS for a number of clinical indications [40]. Validation of TCS with magnetic resonance angiography or digital subtraction angiography in ischemic stroke patients have been made [25–28]. The present study provides a preliminary piece of evidence supporting that TCS is helpful for identifying a subgroup of stroke patients with worse clinical course and poorer outcome, who may benefit from more rigorous preventive and therapeutic measures as has been shown in recent clinical trials [5,6].

There are a number of limitations in the present study. The study subjects were recruited from a multi-center stroke registry, the TSR [20]. Only patients with both extracranial and intracranial vascular sonographic studies were included in this analysis. It is possible that some patients with known etiologies, such as cardioembolism, or with very severe deficit early in clinical course might have been precluded from receiving TCS. Second, the TCS study requires a good acoustic window to identify the MCA. However, patients with older age, particularly in women, may impede the insonation of the intracranial vessels, including the MCA. Lower number of female patients in the present study may reflect a possible gender bias. Third, the vascular studies were often done immediately after the stroke. It is possible that the vascular process during the acute stage might have evolved later. Therefore, some of the patients considered to have MCA stenosis may have recanalized embolus. But the distribution of atrial fibrillation was insignificantly different between groups in Tables 1 and 3. Fourth, ICAS may involve arteries other than the MCA. TCS, focusing on the MCA, does not allow a more comprehensive assessment of different vascular territories. Finally, angiographic study was not widely used in this stroke population to allow confirmation of the validity of TCS results in the present study. Despite these limitations, results presented here are still of value for confirming the prognostic importance of MCA stenosis for a large population of ischemic stroke patients in Taiwan and other Asian countries, and suggest a role for TCS to identify this potentially important marker in patients with ischemic stroke for more assertive measures in stroke prevention.

In conclusion, the frequency of MCA stenosis was 2.3-fold higher than that of extracranial ICA stenosis in ischemic stroke patients in Taiwan. Patients with MCA stenosis were more likely to have DM and hypercholesterolemia and to present with severe neurological deficit with worse outcome. TCS is of practical value for identifying a subgroup of patients who are in need of more rigorous preventive and therapeutic measures. Regarding substantially high prevalence of MCA stenosis in Asian countries, applying TCS to acute ischemic stroke patients in clinical practice may be considered.
